# Optimized Sample Preparation and Microscale Separation Methods for High-Sensitivity Analysis of Hydrophilic Peptides

**DOI:** 10.3390/molecules27196645

**Published:** 2022-10-06

**Authors:** Gábor Tóth, Simon Sugár, Mirjam Balbisi, Balázs András Molnár, Fanni Bugyi, Kata Dorina Fügedi, László Drahos, Lilla Turiák

**Affiliations:** 1 MS Proteomics Research Group, Research Centre for Natural Sciences, Magyar Tudósok Körútja 2, H-1117 Budapest, Hungary; 2 Doctoral School of Pharmaceutical Sciences, Semmelweis University, Üllői út 26, H-1085 Budapest, Hungary; 3 Department of Inorganic and Analytical Chemistry, Budapest University of Technology and Economics, Szt. Gellért tér 4, H-1111 Budapest, Hungary; 4 Hevesy György PhD School of Chemistry, Eötvös Loránd University, Pázmány Péter Sétány 1/A, H-1117 Budapest, Hungary

**Keywords:** mass spectrometry, solid-phase extraction, chromatography, reversed phase, peptide, hydrophilic, cleanup, proteomics, peptidomics, glycopeptides

## Abstract

The optimization of solid-phase extraction (SPE) purification and chromatographic separation is usually neglected during proteomics studies. However, the effects on detection performance are not negligible, especially when working with highly glycosylated samples. We performed a comparative study of different SPE setups, including an in-house optimized method and reversed-phase chromatographic gradients for the analysis of highly glycosylated plasma fractions as a model sample for glycopeptide analysis. The in-house-developed SPE method outperformed the graphite-based and hydrophilic interaction liquid chromatography (HILIC) purification methods in detection performance, recovery, and repeatability. During optimization of the chromatography, peak distribution was maximized to increase the peptide detection rate. As a result, we present sample purification and chromatographic separation methods optimized for the analysis of hydrophilic samples, the most important of which is heavily *N*-glycosylated protein mixtures.

## 1. Introduction

Shotgun proteomics has evolved to be the main technique for deciphering the deep proteome of human and non-human samples. Its clinical importance is unquestionable, especially in the discovery stage of diseases. The analysis of protein glycosylation has emerged to be a substantial part of proteomics-related studies, especially site-specific characterization via the mass spectrometry measurement of tryptic glycopeptides. However, methods are usually optimized for general proteomics workflows or highly carbohydrate-specific applications. Protein glycosylation analysis is challenging due to the structural variability and the highly hydrophilic nature of the sample. Because of the large difference in molecular characteristics, integrating the analysis of samples containing highly glycosylated proteins (thus adding a hydrophilic character) into workflows optimized for general proteomics screening results in sub-optimal analytical performance. This necessitates the constant development of methods for highly glycosylated samples, with secondary considerations of the sample origin as well.

Several different chromatographic techniques have been described for (glyco)proteomics. Reversed-phase (RP) chromatography using C_18_-based packed-bed stationary phase is the most common [1], mainly due to its robustness and easy accessibility as compared to other phases with more specific uses. The state-of-the-art high-performance nanoflow high performance liquid chromatography (nanoHPLC) methods build on the use of 15–50 cm long capillary columns operating with 1–4 h long gradients [2]. The main aims during chromatographic method development for (glyco)proteomics are to maximize peak capacity, the number of detected peptides and proteins, and the sequence coverage of each protein simultaneously [3,4,5]. This can be achieved by careful optimization to maximize the distribution of peaks along the entire elution window. Solid-phase extraction (SPE) purification is inevitable before analysis to eliminate matrix effects and increase the lifespan of the separation column. Similarly, to chromatographic separation, SPE is mainly operated with C_18_-based resins. However, there is a larger variety of available stationary phases on the market. The importance of a sample-type-dependent optimization is not negligible for this step. Temperature, solvent composition during loading and elution, and the type and concentration of ion pairing agents at loading can basically determine the recovery and peptide detection performance [6,7,8]. In cases where the methods are not well optimized for a given sample type, a significant detection loss can be expected, mainly due to insufficient retention during SPE purification and trapping, ion suppression caused by co-elution, and retention time shifts during the first half of the elution window.

For the SPE purification of glycopeptides, mainly C_18_-based [9] resins are used. However, when enrichment is employed at the glycopeptide level, this step is usually skipped, as during the process most of the unwanted matrix components are washed away. For glycopeptide enrichment, hydrophilic interaction liquid chromatography (HILIC) is the most widely used technique. HILIC is a useful tool for the comprehensive characterization of glycoproteins and their glycan isomers [10,11]. Moreover, the potential of the HILIC columns for the isomeric separation of fucosylated and sialylated glycoforms has already been demonstrated [12]. It can provide good separation based on the structure of the glycan side chains. However, it might not be ideal for the separation of the non-glycosylated peptides in the mixture. For these reasons, as an SPE method, HILIC is primarily used for the enrichment of glycans or glycopeptides [13], an example being the cotton-HILIC enrichment of glycans [14,15].

An alternative chromatographic mode in glycopeptide analysis is porous graphitized carbon (PGC) stationary phases, both for purification and separation of hydrophilic peptides. PGC has a unique retention mechanism comprising a combination of hydrophobic interactions, polar interactions of polarizable or polarized groups, and electronic interactions. The use of PGC allows for simplicity, good resolution, repeatability, and recovery. However, these properties are limited when investigating strongly polar components, due to the strong interactions compromising proper elution. Thus, graphite columns are the first choice when short, polar peptides need to be separated [16], up to the point of isomeric separation of different *N*-glycopeptides [17]. Taking these into consideration, the graphite-based methods might complement well RP SPE purification methods, where the most hydrophilic portion of the sample could be lost due to improper binding.

Here we present a detailed comparison of SPE methods for the purification of strongly hydrophilic peptide samples with fractionated plasma as a model sample. Two C_18_-based protocols, two different graphite phases, and a self-packed cotton-HILIC phase were compared in terms of detection and quantitation performance and selectivity. Next, we developed an RP-HPLC gradient maximizing the separation performance of hydrophilic species, thus increasing the detection and quantitation performance of the shotgun glycoproteomic workflow.

## 2. Results and Discussion

### 2.1. Sample Cleanup in the SPE System

**Figure 1:**
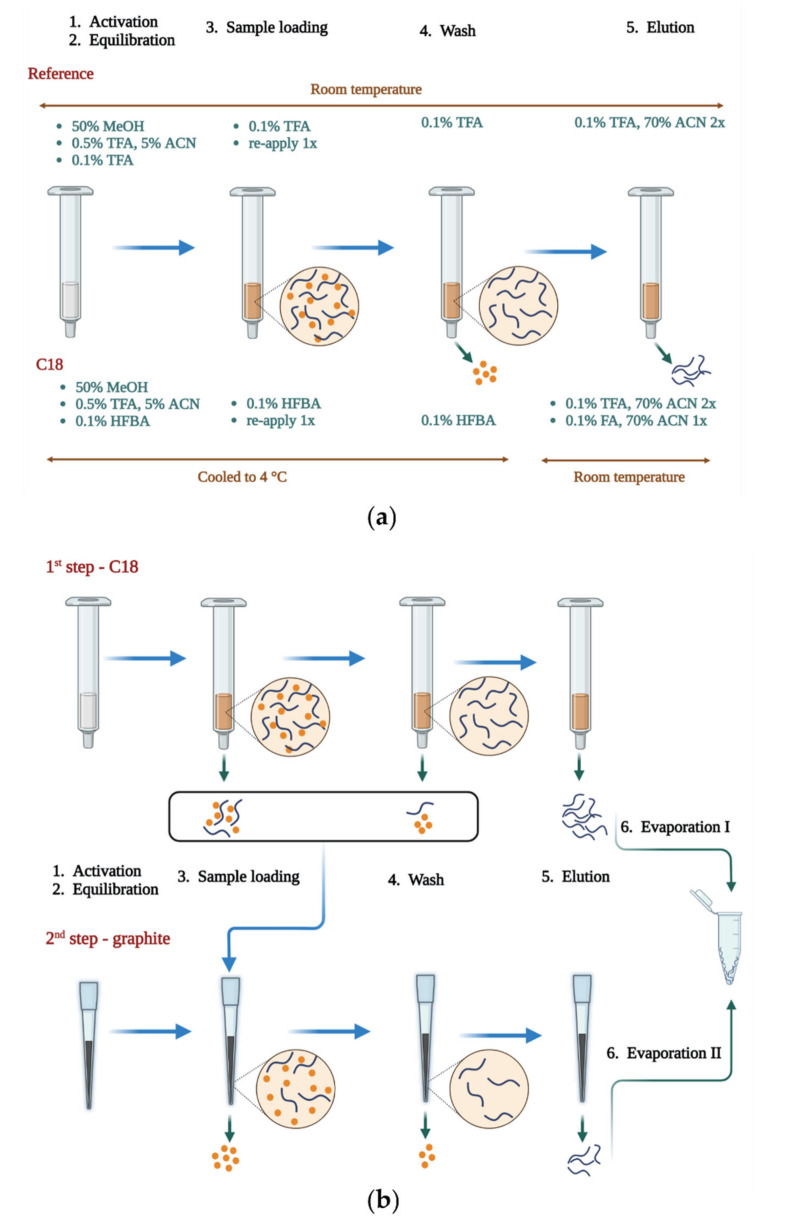
(**a**) Demonstration of differences between the C18 and reference methods. (**b**) Workflow for the combined C18 + graphite methods.

The goal of this Section of the study was to compare our in-house optimized C_18_ protocol (for a detailed protocol, see Section 3.3) developed for the purification of hydrophilic peptide samples to a number of other SPE methods. The C18 method bears differences in several aspects from the reference method: (i) the cartridge and the buffers (except the elution buffer) were cooled to 4 °C instead of room temperature, (ii) the ion pairing reagent for loading and wash was changed to heptafluorobutyric acid (HFBA) instead of trifluoroacetic acid (TFA), and (iii) a third elution step was added with formic acid (FA) instead of TFA to reduce ion pairing effects (Figure 1a).

The evaluation of performance was tested on fractionated plasma samples and was based on multiple characteristics, both qualitative, and quantitative. The tested SPE methods were as follows: C18 (C_18_ sorbent with in-house optimized method for hydrophilic species), TopTip (graphite sorbent), Pierce (graphite sorbent), and Cotton (cotton sorbent with HILIC characteristics). Moreover, two combined methods were tested where the flow-through of the C18 cleanup was further purified with the respective graphite resin (C18 + TopTip and C18 + Pierce). For a visual demonstration of the combined methods, see Figure 1b. As a reference method, the C_18_ sorbent using the manufacturer’s protocol was chosen. Each method was carried out in triplicate using 1 µg of fractionated plasma (for exact protocols, see Section 3.3).

**Figure 2:**
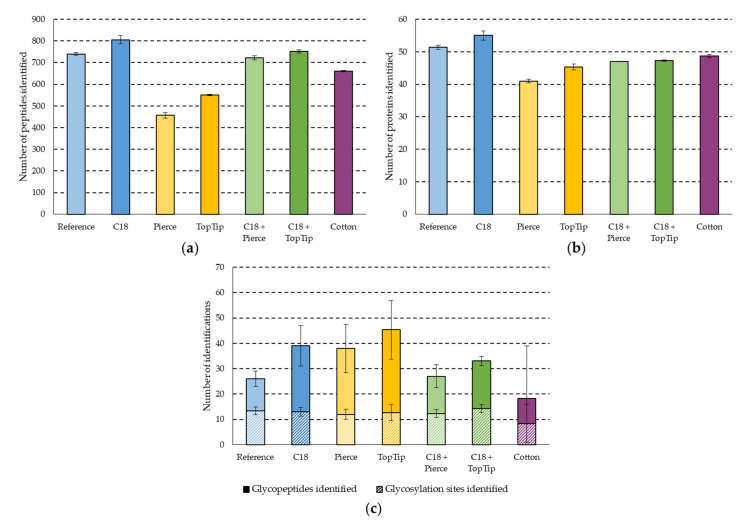
The number of peptides (**a**), proteins (**b**), and glycopeptides and glycosylation sites (**c**) detected with each SPE clean up method. Samples were measured in triplicate; error bars represent standard deviation.

First, the performance of the seven SPE methods was compared using qualitative measures: the number of proteins, peptides, glycopeptides, and glycosylation sites detected. These are shown in Figure 2.

The performance of the different SPE methods was markedly different. The C18 method showed the best performance regarding peptide detection, with more than 800 peptides detected on average (Figure 2a). The other C_18_-based methods were slightly worse (between 700 and 750 peptides detected), while the Pierce, TopTip, and Cotton methods performed significantly worse (less than 500, 600, and 700, detections respectively). These differences, however, were much smaller at the protein detection level, where the difference between the best and worst methods was only 25% (C18 and Pierce methods) compared to 43% for peptides. The C18 showed better performance than the Cotton and both graphite-based (Pierce, TopTip) sorbents (Figure 2b); on average, 41–49 proteins were detected with other sorbents, while this was 55 for the C18 method. It also showed a ca. 10% detection gain as compared to the reference method with the same sorbent. These small differences in protein detection are attributed to the relatively low complexity of the samples.

Next, we combined the C18 method with the graphite-based setups to see if an additional gain could be achieved by purifying the flow-through and combining the two elution fractions (Figure 1b). Both combined methods showed inferior performance to the C18 and reference methods. One possible explanation for this is that solvent evaporation is carried out twice using the combined method, thus decreasing peptide recovery after purification (Figure 2a). Note that 20–30% sample loss is expected during solvent evaporation with a heated vacuum centrifuge (unpublished data). However, this loss is strongly influenced by the organic solvent content, the volume, and thus the time of evaporation. Higher organic solvent content results in a larger interaction surface for non-specific binding with the tube walls, higher organic solvent content elevates the possibility of droplets escaping the tube, and longer times facilitate degradation of peptides and permanent interactions with tube walls. In our method, we evaporated solvents after the first step, and then the elution fraction of the second step was pipetted in the same Eppendorf tube and a second evaporation took place. Thus, two evaporation steps were performed for both parts of each sample in the combined methods, in contrast to the reference or the C18 methods, where only one drying down/reconstitution step is performed between the purification and the measurement. The increase in sample loss caused by the evaporation is balanced by the gain attributable to the second purification step. In the presented combinations, the evaporation sample loss was larger than the gain from the second purification, thus decreasing the number of detected peptides and proteins. 

Although the graphite-based SPE methods performed worst for the clean-up of non-glycosylated species, they were among the best for glycopeptides (Figure 2c). The TopTip method was significantly better than any other method (on average over 45 glycopeptides were detected), while the Pierce and the C18 methods also showed good performance (38 and 39 detections, respectively). The Cotton method performed the worst (60% fewer detections than the TopTip method), contrary to our expectations based on its frequent use as a glycopeptide enrichment method. The number of detected glycosylation sites was highly similar for all methods except for the Cotton method, which is presumably due to the low reproducibility of the method (indicated by the high standard deviation in Figure 2c).

The assessment of possible differences in selectivity between the different SPE methods was carried out by the comparison of detected glycosylation sites; glycan types for glycopeptides; and peptide length, hydrophobicity (Grand Average of Hydropathy, GRAVY scores), and isoelectric point distributions for peptides.

Regarding glycopeptides, no differences in selectivity could be detected. The glycan types detected were mostly bi-, tri-, and tetra-antennary complex types for each method, and all of them contained both non-sialylated and highly-sialylated variants, which suggests no major differences in selectivity towards glycans (Table A1).

**Figure 3:**
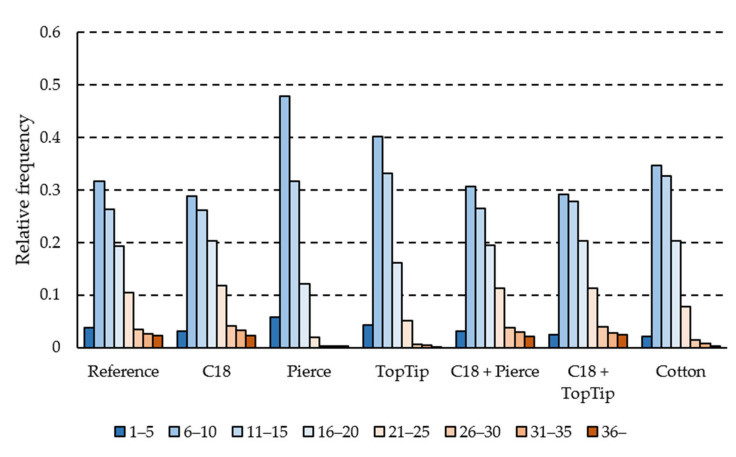
Peptide length distributions of detected peptides for the SPE cleanup methods.

**Figure A1:**
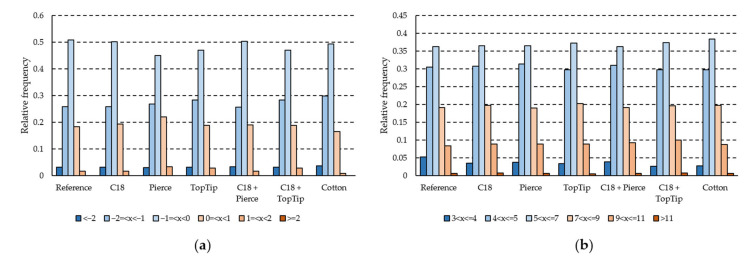
(**a**) GRAVY score distribution of peptides detected with each SPE method. (**b**) Isoelectric point distributions of peptides detected with each SPE method.

Similarly, peptide hydrophobicity (described by the GRAVY score) and isoelectric point distributions did not reveal significant differences in selectivity towards peptide backbones (Figure A1). GRAVY score and isoelectric point distributions showed excellent correlation between all methods (mean correlation coefficient of 0.995 and 0.998, respectively). However, peptide length distributions were different between the C_18_-containing sorbents and the Pierce, TopTip, and Cotton methods (Figure 3).

The shift in the peptide length distribution towards smaller peptides for the Pierce, TopTip, and (to a lesser extent) the Cotton methods (Figure 3), combined with the lower peptide detection numbers (Figure 2a), suggests that these methods are selective towards shorter peptides, and a significant number of larger peptides were lost. For the graphite-based sorbents, this is in line with their retention characteristics and suggests that the retention is dominated by polar interactions in this solvent system. This hypothesis is supported by the fact that peptide length distributions were heavily affected, while GRAVY score (average hydrophobicity normalized to peptide length) distributions were not (Figure A1).

**Figure A2:**
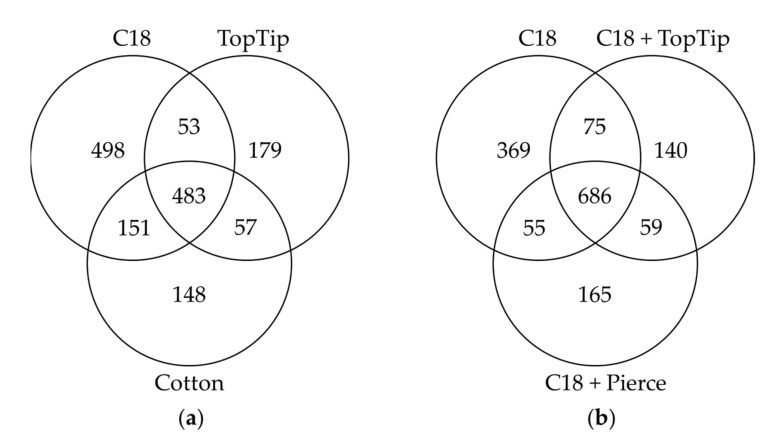
(**a**) Peptide detection overlap for the C18, TopTip, and Cotton methods. (**b**) Peptide detection overlap for the C18 and the two combined methods.

Overall selectivity differences between the methods were addressed by comparing all the detected peptides on Venn diagrams. When comparing the C18 method with the TopTip and Cotton methods, we could conclude that only a minor selectivity difference could be observed. More than 70% of all peptides could be detected using the C18 method, but more than 30% could not be detected while using either TopTip or Cotton (Figure A2a). On the other hand, the unique detections with the TopTip and Cotton methods were around 10% of all peptides, which is comparable to the variability attributed to the data-dependent acquisition mode. Furthermore, there was no significant additional selectivity gain by using another type of stationary phase combined with the C18 method (Figure A2b).

**Figure 4:**
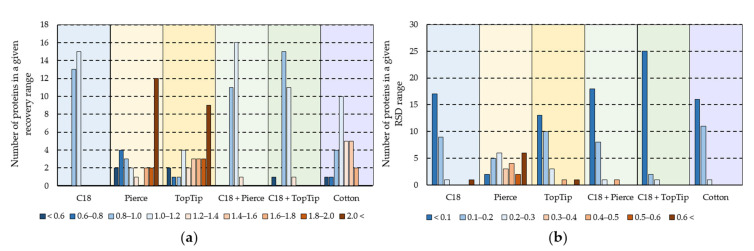
(**a**) Protein recovery value distributions relative to the reference method; (**b**) Protein LFQ intensity RSD value distributions for the six SPE methods.

Next, the performance of the different SPE methods was compared from a quantitative aspect. For this comparison, MaxQuant LFQ (Label-Free Quantitation) intensity values were used, and recovery values were calculated for each method relative to the reference method for proteins quantified in all the samples (28 in total). The recovery value and LFQ intensity relative standard deviation (RSD) distributions are presented in Figure 4.

The recovery value distributions (Figure 4a) show great variation between the different methods. For the C_18_-containing methods, most recovery values were between 0.8 and 1.2. However, for the graphite sorbents and the Cotton method, they showed a much wider distribution, which suggests uncontrolled binding and elution performance for the different peptides. The RSD values also varied greatly (Figure 4b) between the different methods. The C_18_-containing methods clearly showed superior repeatability compared to other methods, with the majority of RSD values under 0.1. Overall, the quantitative comparison suggests that there are differences in selectivity between the different methods (especially between the C_18_-containing and the other three methods) and that C_18_-based methods are more suitable for comparative proteomics due to their excellent repeatability.

In summary, the in-house optimized C18 method outperformed all other methods for the cleanup of heavily glycosylated samples in terms of peptide and protein detection and quantitation. This method has proven excellent utility in the analysis of other sample types as well in our laboratory, such as FFPE tissues [18], cell lines, and extracellular vesicles (unpublished data). The graphite-based methods showed slightly different selectivity than the C_18_-based methods, and the TopTip method was best for the detection of glycopeptides. On the other hand, the combined (C_18_ and graphite) methods showed similar behavior to the one-step C18 method, which implies that the addition of different retention mechanisms did not improve performance. Finally, the Cotton method showed poor overall performance except for good repeatability for quantitation. The performance of the different SPE methods is summarized in Table 1.

### 2.2. Reversed-Phase Gradient Separation

During the gradient development, we compared the performance of five different gradients. The gradient programs for the distinct gradients are summarized in Section 3.4. As the initial slope was presumed to have a crucial effect on the separation of the hydrophilic region, we designed methods from 0.21 to 0.51% acetonitrile/min initial slopes (Table 2).

**Figure 5:**
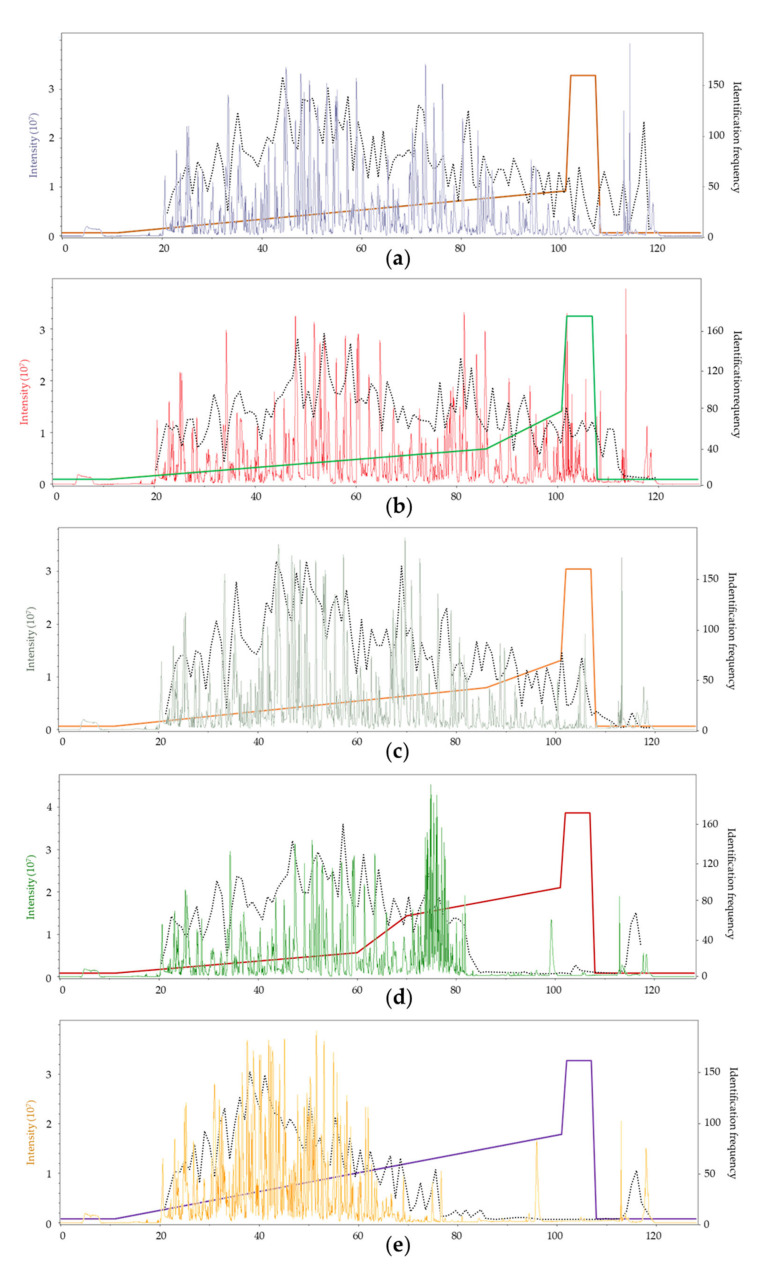
Representative chromatograms and the peptide detection rate (dotted line) obtained with the five different gradients. The gradient slopes are overlaid in the background (without a corresponding scale). (**a**) Lin 4–27; (**b**) 2step 4-20-40; (**c**) 2step 4-25-40; (**d**) 3step 4-15-35-50; and (**e**) Lin 4–50.

The most important factor influencing the repeatability and detection performance of the chromatographic method is the distribution of peaks throughout the elution window. It depends on both the initial and average slope of the gradient. The 2step 4-20-40 method provided perfect peak distribution in the whole elution window (Figure 5b). The peak distributions using the other two lower-slope methods (Lin 4–27 and 2step 4-25-40) were also close to ideal. However, the higher initial slopes (0.256 and 0.280, respectively) caused the decrease of retention times generally, thus generating a time window scarce in peaks (95–110 min, Figure 5a,c). This difference is well-reflected in the detection rate; more peptides were detected using the 2step 4-20-40 method after 90 min, especially in the 110–120 min region, than with the other two above-mentioned ones. This effect is even more remarkable when looking at the two gradients with a larger average slope (Figure 5d,e). The shallow first gradient step in the 3step 4-15-35-50 method resulted in ideal peak distribution in the first 60 min, but the high second slope caused a stacked peak distribution between 70 and 80 min and a region scarce in peaks between 85 and 110 min. Finally, using a large linear slope in the Lin 4–50 method resulted in the elution of most of the peptides in the first 70 min in a visibly stacked manner, causing a region lacking peaks after 70 min. This unfavorable peak distribution resulted in the lack of detected peptides after 90 min and 78 min, respectively. The ion suppression caused by the stacked peak distribution made peptide detection less effective between 70 and 80 min for the 3step 4-15-35-50 method and between 50 and 80 min for the Lin 4–50 method.

The number of detected peptides ranges from 896 to 971 with the use of the different gradients (Table 3). Generally, gradients with a lower overall average slope (Lin 4–27, 2step 4-20-40, and 2step 4-25-40) resulted in better peptide detection numbers due to the near-optimal distribution of peaks through the elution window. 

Peptide and glycopeptide detections up until 90 min are in good correlation with the effect of the average slope until that point. The three shallower gradients (2step 4-20-40, Lin 4–27, and 2step 4-25-40 in increasing order of slope) showed a decreasing trend with increasing slope due to co-elution and ion suppression of some components. The two higher-slope gradients resulted in higher detection rates that are almost identical since they had the same average slope, and almost all the components eluted in 90 min in both cases. Note that in the case of the shallower gradients, on average 102–169 peptides and up to 21 glycopeptides were detected in the last part of the elution window, while practically no additional detection happened in that Section when using the higher-slope gradients. Similar trends could also be observed when analyzing the detection rates until 60 min. 

Surprisingly, the differences in peptide and glycopeptide detection were not reflected in protein and glycosite detection. The methods with a higher average slope allowed for slightly fewer detected proteins and slightly more glycosites, but the differences were not significant (Table 3).

Selectivity and quantitation performance can also be affected by the distribution of peaks in the different (hydrophilic and hydrophobic) regions of the chromatogram. However, these differences are inherently smaller than those seen with different sorbents for SPE. Small differences were observed with regard to the peptide length and GRAVY score distribution of peptides. Regarding quantitation, a gain of only 3% in the average peak areas was observed when using the optimal gradient as compared to the 3step 4-15-35-50 method. Additional information is provided in Appendix A.

In summary, gradients with shallower starting conditions resulted in better peak distribution not only in the first part but throughout the whole elution window. The 2step 4-20-40 method provided the best peak distribution and quantitation performance as well as repeatability in all the investigated means of measure (Table 4).

## 3. Materials and Methods

### 3.1. Materials

All chemicals used were HPLC-MS grade. Acetonitrile (ACN), water, acetone, formic acid (FA), ammonium-bicarbonate, and heptafluorobutyric acid (HFBA) were purchased from Merck (Darmstadt, Germany). Trifluoroacetic acid (TFA), dithiothreitol, and iodoacetamide were obtained from Thermo Scientific (Unicam, Budapest, Hungary). Methanol was purchased from VWR International (Debrecen, Hungary) and RapiGest was obtained from Waters (Budapest, Hungary).

### 3.2. Sample Preparation

Depleted and pooled human plasma samples were fractionated as described before [19]. In brief, a Poros R2 column was used, with a column temperature of 65 °C and a flow rate of 1 mL/min. The gradient started with 20% B and continued for 0.7 min, followed by a 15 min long gradient from 20 to 70% solvent B. Solvent A was water containing 0.07% (*v/v*) trifluoroacetic acid and solvent B was acetonitrile containing 0.07% (*v/v*) trifluoroacetic acid. The fraction between 2.0 and 2.5 mL was collected manually as a heavily glycosylated fraction. These fractions from five pooled human plasma samples were pooled for each set of experiments and then dried down with SpeedVac (miVac Duo Concentrator, Genevac Ltd., Ipswich, Suffolk, UK).

Digestion was carried out as described before [20,21]. The sample was dissolved in 10 μL of 8 M urea in 50 mM ammonium bicarbonate. DTT was added at a final concentration of 5 mM and incubated at 37 °C for 30 min. Alkylation was performed in the dark at room temperature for 30 min in the presence of 10 mM IAA. Samples were diluted 10-fold with 50 mM ammonium bicarbonate and 1 μL of a 10 ng/μL Trypsin/Lys-C mix (Promega, Madison, WI, USA) was added and incubated at 37 °C for 80 min. Next, 1 μL of 40 ng/μL Trypsin (Promega, Madison, WI, USA) was added and the samples were incubated for another 2 h. Digestion was quenched by the addition of 1 μL of formic acid. Samples were separated into aliquots containing 1 μg of protein, then dried down and stored at -20 °C until further use.

### 3.3. Solid-Phase Extraction Cleanup

Sample cleanup was performed with spin tip SPE systems using centrifugation at 2500× *g* for 1 min. The sample loading, wash, and elution conditions used for the SPE optimization are summarized in Table 5. For the gradient optimization part, C18 SPE cleanup was used. After cleanup, all the samples were dried down and stored at −20 °C until reconstitution for measurement.

When applying the combined methods C18+Pierce and C18+TopTip, the flow-through of the C18 method at sample loading and wash were collected, dried down, and loaded to a Pierce or a TopTip phase, respectively. The elution fractions were added to the dried elution fraction of the C18 cleanup and dried down once again together. Therefore, the sample was subjected to solvent evaporation twice: the sample loading and wash fraction of the C18 step before the second loading to the graphite phase and after the elution from it, while the elution fraction of the C18 step was added after the elution and then after the addition of the elution fraction of the second (graphite) step.

### 3.4. Reversed-Phase Chromatographic Separation

Samples were dissolved in injection solvent (98% H_2_O, 2% ACN, and 0.1% FA) and were subjected to nanoLC-MS/MS analysis using a Dionex Ultimate 3000 RSLC nanoHPLC (Dionex, Sunnyvale, CA, USA). Trapping was performed on an Acclaim PepMap100 C18 (5 µm, 100 µm × 20 mm, Thermo Fisher Scientific, Waltham, MA, USA) trap column with 0.1% TFA + 0.01% HFBA (H_2_O) as the transport liquid. Peptides were separated on an Acquity M-Class BEH130 C_18_ analytical column (1.7 µm, 75 µm × 250 mm Waters, Milford, MA, USA) using the gradients listed in Table 6. Solvent A was 0.1% FA in H_2_O, Solvent B was 0.1% FA in ACN, the flow rate was 300 nL min−1, and the column temperature was 48 °C. The method 4-25-40 was used for the separation during the SPE optimization phase with 600 ng injected amounts.

### 3.5. Mass Spectrometry Analysis

The nanoHPLC was coupled to a Bruker Maxis II QTOF (Bruker Daltonik GmbH, Bremen, Germany) via the CaptiveSpray nanoBooster ionization source (0.1% FA in ACN as booster liquid). Spectra were collected using a fixed cycle time of 2.5 sec and the following scan speeds: MS spectra at 3 Hz, while CID was performed on multiply charged precursors at 16 Hz for abundant ones and at 4 Hz for low-abundance ones. Internal calibration was performed by infusing sodium formate and data were automatically recalibrated using the Compass Data Analysis (v4.3; Bruker Daltonik GmbH, Bremen, Germany) software.

### 3.6. Data Analysis

Protein, peptide, and glycopeptide detection was performed using Byonic [22] (version 4.2.10) on a SwissProt Homo Sapiens database, with the maximum number of missed cleavages set at 2. Carbamidomethylation was set as a fixed modification, and methionine oxidation, acetylation, and deamidation were set as variable modifications. Protein FDR was controlled at 1%. For glycopeptide detection, the “N-glycan 182 no multiple fucose” built-in database was used. Protein quantitation was performed using MaxQuant [23] (version 1.6.17.0) on a SwissProt Homo Sapiens database with the maximum number of missed cleavages set at 2. Carbamidomethylation was set as a fixed modification, and methionine oxidation, acetylation, and deamidation were set as variable modifications. At all levels, FDR was controlled at 1%. ‘Match between runs’ was used with a 2 min window. Further important software settings are included in Table A2 and Table A3, respectively. The isoelectric points were calculated using the IPC—Isoelectric Point Calculator by Kozlowsky [24], and GRAVY scores [25] were calculated in Microsoft Excel. The data were then processed in Microsoft Excel, which was then used for all further calculations. Data visualization was performed using Microsoft Excel and PowerPoint. The graphical abstract and Figure 1 were created with Biorender.com.

## 4. Conclusions

In mass spectrometry-based proteomics, the effect of the sample cleanup and chromatographic separation is frequently overlooked. However, a significant gain in performance can be attributed to carefully optimized methods. We presented optimized methods for both the SPE cleanup and the chromatographic separation of highly glycosylated samples using fractionated human plasma as a model.

We compared seven different SPE methods, from which the in-house optimized C18 cleanup method showed excellent performance regarding protein and peptide detection and quantitation, as well as similar performance to the best-performing TopTip graphite phase for glycopeptide detection. This method has already been applied to several samples from various origins and extents of glycosylation and provided excellent results. The possible performance gain from the use of combined C_18_ and graphite phases was also tested. However, the optimized C18 method was found superior.

Using a well-optimized gradient, up to 8.4% gain was observed in the peptide detection numbers compared to a standard linear gradient. The good distribution of peaks in the first half of the elution window was particularly useful. These differences were not reflected in glycosite and protein detection, and selectivity is only moderately influenced by the selection of the gradient. However, the performance of quantitation is also affected to a minor extent. Using the developed method (2step 4-20-40), the detection and quantitation numbers are optimal along with experiencing the smallest standard deviations.

## Figures and Tables

**Table 1:** Summary of the performance of the different SPE methods discussed. (+++ means best, -- means worst performance characteristics, relative to the other methods).

Purification Method	Reference	C18	Pierce	TopTip	C18 + Pierce	C18 + TopTip	Cotton
Peptide detection	++	+++	--	-	++	++	+
Protein detection	++	+++	--	-	+	+	+
Glycopeptide detection	-	++	++	+++	-	+	--
Glycosite detection	+++	+++	+++	+++	+++	+++	--
Quantitation		+++	-	-	+++	++	-
Repeatability		++	--	+	++	+++	++
Selectivity for shorter peptides	--	--	+++	+++	--	--	+
Overall performance	++	+++	-	+	++	++	--

**Table 2:** The steepness of the gradients between the various steps and the average slope for the whole elution window. ACN: acetonitrile.

Gradient Program	Lin 4–27	2step 4-20-40	2step 4-25-40	3step 4-15-35-50	Lin 4–50
Step 1 (%ACN/min)	0.256	0.213	0.280	0.225	0.511
Step 2 (%ACN/min)	-	1.33	1.00	2.00	-
Step 3 (%ACN/min)	-	-	-	0.485	-
Average slope (%ACN/min)	0.256	0.400	0.400	0.511	0.511

**Table 3:** Summary of detection values for different species for the compared gradient programs.

Gradient	Lin 4–27	2step 4-20-40	2step 4-25-40	3step 4-15-35-50	Lin 4–50
Peptide detections	920 ± 13	971 ± 21	937 ± 16	890 ± 21	896 ± 16
Glycopeptide detections	46 ± 2	44 ± 1	49 ± 7	40 ± 2	44 ± 3
Glycosite detections	15 ± 0	16 ± 1	15 ± 2	17 ± 3	17 ± 1
Peptide detections under 90 min	810 ± 8	869 ± 20	768 ± 17	877 ± 20	879 ± 8
Glycopeptide detections under 90 min	43 ± 2	38 ± 1	28 ± 5	40 ± 2	44 ± 3
Peptide detections under 60 min	574 ± 1	610 ± 18	523 ± 19	537 ± 13	765 ± 5
Glycopeptide detections under 60 min	8 ± 2	7 ± 2	3 ± 2	6 ± 3	37 ± 3
Protein detections	120 ± 6	126 ± 3	122 ± 7	124 ± 6	126 ± 4
Average peptide/protein	8 ± 0	8 ± 0	8 ± 0	7 ± 0	7 ± 0

**Table 4:** Overall evaluation of the gradient programs presented. (+++ means best, --- means worst performance characteristics, relative to the other methods).

Property	Lin 4–27	2step 4-20-40	2step 4-25-40	3step 4-15-35-50	Lin 4–50
Peak distribution	++	+++	++	--	---
Peptide detection	+	+++	++	-	-
Protein detection	++	+++	++	+++	+++
Glycopeptide detection	++	++	+++	-	++
Repeatability	++	+++	++	++	+++
Selectivity				shorter peptides	
Quantitation	-	+++	+	---	+
Overall performance	+	+++	++	--	-

**Table 5:** Stationary phases and solvent systems used for finding the optimal SPE method for cleanup.

Protocol	Reference	C18	Pierce	TopTip	Cotton
Stationary phase type	C18	C18	graphite	graphite	self-packed cotton
Activation	200 µL 50% MeOH, twice	200 µL 50% MeOH, twice	100 µL 80% ACN 0.1% TFA, twice	100 µL 80% ACN 0.1% TFA, twice	50 μL 60% ACN
Equilibration I	200 µL 0.5% TFA 5% ACN, twice	200 µL 0.5% TFA 5% ACN, twice	100 µL water, twice	100 µL water, twice	50 μL 1% TFA, 98% ACN
Equilibration II	200 µL 0.1% TFA, twice	200 µL 0.1% HFBA, twice	-	-	-
Sample loading	50 µL 0.1% TFA, FT applied once more	50 µL 0.1% HFBA, FT applied once more	50 µL water, then FT applied once more	50 µL water, then FT applied once more	30 μL 1% TFA, 95% ACN, then FT applied twice more
Wash	100 µL 0.1% TFA, twice	100 µL 0.1% HFBA, twice	50 µL water, thrice	50 µL water, thrice	50 μL 1% TFA, 95% ACN
Elution I	50 µL 70% ACN 0.1% TFA, twice	50 µL 70% ACN 0.1% TFA, twice	50 µL 40% ACN 0.05% TFA, thrice	50 µL 40% ACN 0.05% TFA, thrice	10 μL 0.1% FA at 40 °C
Elution II	-	50 µL 70% ACN 0.1% FA, once	-	-	-

FT: flow-through, FA: formic acid, TFA: trifluoracetic acid, HFBA: heptafluorobutyric acid, ACN: acetonitrile. In the case of the C18 method, the cartridge and all the solvents except that for elution were thermostated at 4 °C.

**Table 6:** Gradient programs used during the optimization of the chromatographic method.

Gradient Name	Lin 4–27		2step 4-20-40		2step 4-25-40		3step 4-15-35-50		Lin 4–50	
	time	B%	time	B%	time	B%	time	B%	time	B%
	0	4	0	4	0	4	0	4	0	4
	11	4	11	4	11	4	11	4	11	4
	101	27	86	20	86	25	60	15	101	50
	102	90	101	40	101	40	70	35	102	90
	107	90	102	90	102	90	101	50	107	90
	108	4	107	90	107	90	102	90	108	4
	128	4	108	4	108	4	107	90	128	4
			128	4	128	4	108	4		
							128	4		

**Figure A3:**
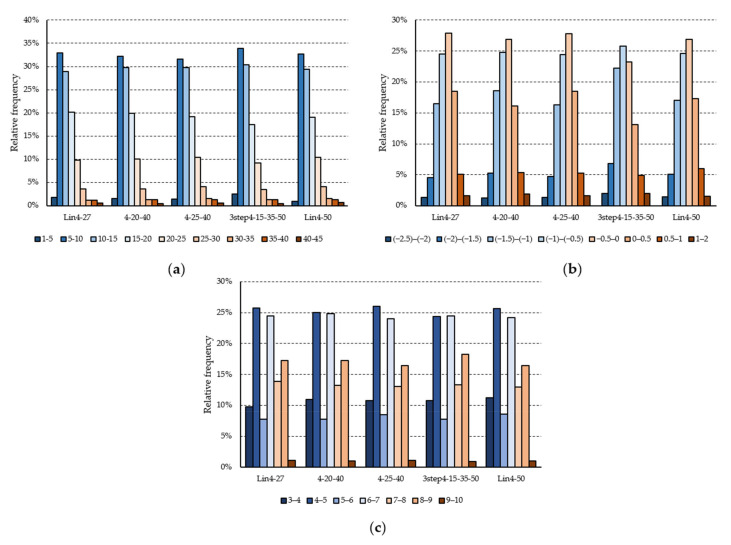
Detection selectivity with the presented gradients. (**a**) Peptide length distribution, (**b**) GRAVY score distribution, and (**c**) Isoelectric point distribution. Note that the last bar in panel (**b**) denotes a different range than all the other increments.

**Figure A4:**
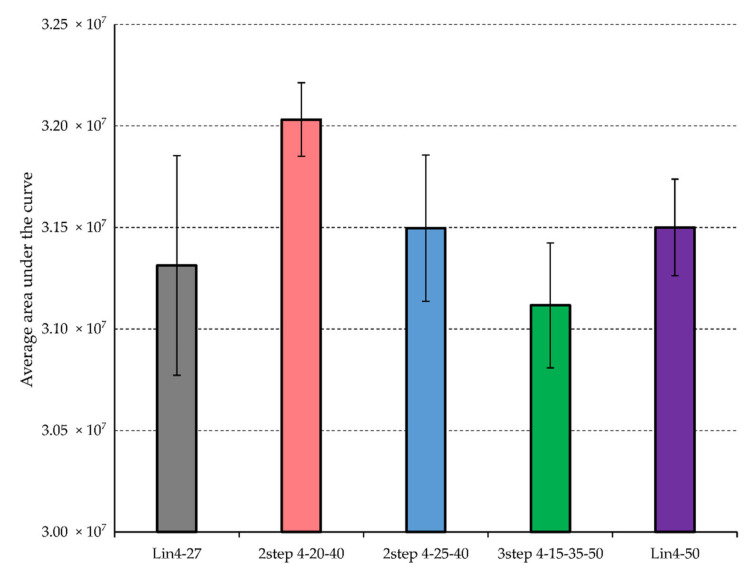
Average LFQ values of quantified proteins by using the presented gradient methods.

## Data Availability

Experimental data has been submitted to the MassIVE data repository (https://massive.ucsd.edu/ProteoSAFe/static/massive.jsp, accessed on 15 August 2022) with the ID: MSV000090135.

## Sample Availability

Samples used in the study are available from the authors upon request.

## Appendix A

Characterization of selectivity of the tested SPE methods.

**Table A1:** The different glycans detected (attached to the glycopeptide backbone) with each SPE method. N: N-acetyl hexosamine, H: hexose, F: fucose, S: sialic acid.

Glycan Type	Reference	C18	Pierce	TopTip	C18 + Pierce	C18 + TopTip	Cotton
Non-complex type	-	N3H4S1	-	N2F1	-	-	N3H4S1
		N3H6		N3H4S1			
Bi-antennary complex type	N4H5	N4H5	N4H5	N4H5	N4H5	N4H5	N4H5
	N4H5F1S1	N4H5F1	N4H5F1	N4H5F1	N4H5F1S1	N4H5F1S1	N4H5F1S1
	N4H5F1S2	N4H5F1S1	N4H5F1S1	N4H5F1S1	N4H5F1S2	N4H5F1S2	N4H5F1S2
	N4H5S1	N4H5F1S2	N4H5F1S2	N4H5F1S2	N4H5S1	N4H5S1	N4H5S1
	N4H5S2	N4H5S1	N4H5S1	N4H5S1	N4H5S2	N4H5S2	N4H5S2
		N4H5S2	N4H5S2	N4H5S2	N4H6F1S1		N4H6F1S1
		N4H6F1					
		N4H6F1S1					
Tri-antennary complex type	N5H4S2	N5H3F1S1	N5H5S2	N5H5F1S1	N5H4S1	N5H6F1S2	N5H4S2
	N5H6F1S2	N5H4S1	N5H6F1S1	N5H6	N5H5S1	N5H6F1S3	N5H6F1S3
	N5H6S2	N5H4S2	N5H6F1S2	N5H6F1	N5H6F1S2	N5H6S1	N5H6S2
	N5H6S3	N5H5F1	N5H6F1S3	N5H6F1S1	N5H6S1	N5H6S2	N5H6S3
		N5H5F1S1	N5H6S1	N5H6F1S2	N5H6S2	N5H6S3	
		N5H5F1S2	N5H6S2	N5H6F1S3	N5H6S3		
		N5H6F1S2	N5H6S3	N5H6S1			
		N5H6F1S3		N5H6S2			
		N5H6S1		N5H6S3			
		N5H6S2					
		N5H6S3					
Tetra-antennary (or larger) complex type	N6H7F1S4	N6H7S1	N6H7F1S2	N6H6F1	N6H7F1S2	N6H7F1S2	N6H7F1S3
	N6H7S1	N6H7S2	N6H7F1S3	N6H6F1S1	N6H7F1S3	N6H7F1S3	N6H7F1S4
	N6H7S2	N6H7S3	N6H7S1	N6H7	N6H7S1	N6H7F1S4	N6H7S1
	N6H7S3	N6H7S4	N6H7S2	N6H7F1S1	N6H7S2	N6H7S1	N6H7S2
	N6H7S4	N7H8F1S2	N6H7S3	N6H7F1S2	N6H7S3	N6H7S2	N6H7S4
			N6H7S4	N6H7F1S3	N6H7S4	N6H7S3	
			N7H8S1	N6H7S1	N7H4F1	N6H7S4	
			N9H6	N6H7S2			
				N6H7S3			
				N6H7S4			
				N7H8F1S3			

The distribution of peaks in different (hydrophilic and hydrophobic) regions of the chromatogram can also affect selectivity; however, these differences are inherently smaller than those seen with different sorbents for SPE. Regarding the length of the detected peptides, all gradients showed practically the same distribution. The 3step 4-15-35-50 method showed a minor difference from the others: there was a 3.3–4.1% higher relative frequency of peptides consisting of a maximum of 15 amino acids (Figure A3a). The same, but more remarkable, differences are reflected in the GRAVY score distributions as well. Using the 3step 4-15-35-50 method, the relative frequency of the hydrophilic peptides (GRAVY < −0.5) was 7–10%, mainly making up for a loss in moderately hydrophobic (GRAVY −0.5–0.5) peptides (Figure A3b). These facts represent a loss in the detection of the longer (thus more hydrophobic) peptides due to the co-elution in the higher retention time regions. The used gradient, however, did not affect the isoelectric point distribution of the detected peptides (Figure A3c).

Next, we addressed the quantitative performance of the gradients. Inefficient peak distribution causes co-elution of the components, thus resulting in uncontrolled ion suppression of the peptides. We considered the average LFQ values of all the quantified proteins as a suitable measure for this. As is seen in Figure A4, the differences are moderate among the different gradients; however, the results are in correlation with the peak distribution. The largest average LFQ values and smaller standard deviations were obtained using the 2step 4-20-40 method, while the smallest LFQ values corresponded to the 3step 4-15-35-50 method. Contrary to our expectations, the smallest repeatability was obtained using the Lin 4–27 method.

**Table A2:** Most important parameters of the Byonic database search.

Rule	Value
Maximum number of missed cleavages	2
Precursor tolerance	10.0 ppm
Fragment tolerance	frag:qtof_hcd 20.0 ppm
Protein FDR cutoff	1%
Common modifications max	1
Rare modifications max	1
Fixed modifications:	Carbamidomethyl (C)
Variable modifications:	Deamidation (NQ); Oxidation (M); Acetylation (N-term)
Glycan database:	Mammalian 182 no multiple fucose
Product version	PMI-Byonic-Com: v4.2.10

**Table A3:** Most important parameters of the MaxQuant database search.

Parameter	Value
Version	1.6.17.0
PSM FDR	0.01
Protein FDR	0.01
Min. peptide length	7
Peptides used for protein quantification	Razor
Match between runs	TRUE
Matching time window [min]	2
Alignment time window [min]	20
Max. peptide mass [Da]	4600
Razor protein FDR	TRUE
MS/MS tol. (TOF)	40 ppm
Fixed modifications	Carbamidomethyl (C)
Variable modifications	Deamidation (NQ); Oxidation (M); Acetylation (N-term)
